# Osteogenic Differentiation of hDPSCs on Biogenic Bone Apatite Thin Films

**DOI:** 10.1155/2017/3579283

**Published:** 2017-10-22

**Authors:** Michele Bianchi, Alessandra Pisciotta, Laura Bertoni, Matteo Berni, Alessandro Gambardella, Andrea Visani, Alessandro Russo, Anto de Pol, Gianluca Carnevale

**Affiliations:** ^1^Rizzoli Orthopaedic Institute, NanoBiotechnology Laboratory, Via di Barbiano 1/10, 40136 Bologna, Italy; ^2^Department of Biomedical, Metabolic and Neural Sciences, University of Modena and Reggio Emilia, Via Campi 287, 41125 Modena, Italy; ^3^Rizzoli Orthopaedic Institute, Laboratory of Biomechanics and Technology Innovation, Via di Barbiano 1/10, 40136 Bologna, Italy

## Abstract

A previous study reported the structural characterization of biogenic apatite (BAp) thin films realized by a pulsed electron deposition system by ablation of deproteinized bovine bone. Thin films annealed at 400°C exhibited composition and crystallinity degree very close to those of biogenic apatite; this affinity is crucial for obtaining faster osseointegration compared to conventional, thick hydroxyapatite (HA) coatings, for both orthopedics and dentistry. Here, we investigated the adhesion, proliferation, and osteogenic differentiation of human dental pulp stem cells (hDPCS) on as-deposited and heat-treated BAp and stoichiometric HA. First, we showed that heat-treated BAp films can significantly promote hDPSC adhesion and proliferation. Moreover, hDPSCs, while initially maintaining the typical fibroblast-like morphology and stemness surface markers, later started expressing osteogenic markers such as Runx-2 and OSX. Noteworthy, when cultured in an osteogenic medium on annealed BAp films, hDPSCs were also able to reach a more mature and terminal commitment, with respect to HA and as-deposited films. Our findings suggest that annealed BAp films not only preserve the typical biological properties of stemness of, hDPSCs but also improve their ability of osteogenic commitment.

## 1. Introduction

For over 20 years in the orthopedic and dental field, metal implants—intended to mechanically interlock and biologically integrate with the host bone tissue—have been routinely coated with bioactive calcium phosphate films in order to overcome their intrinsic bioinertness [1]. Hydroxyapatite (HA) has been far apart the primary choice to fulfill this aim, due to a generally accepted similarity with the inorganic phase of bone [2]. Owing to low-cost, high deposition rate and the possibility to obtain highly porous coatings, commercial HA coatings are currently realized almost exclusively by plasma spraying methods [2].

However, even if many *in vivo* animal studies corroborated the evidence of a better ability of HA-coated metallic implants to promote bone regeneration compared to uncoated ones [3], the results of several long-term human trials failed to conclusively demonstrate a real clinical advantage of coated implants [4]. Moreover, detrimental failure of sprayed HA coatings has been pointed out [5], mainly related to interface delamination, fatigue, or occurrence of cracks.

Because of these issues, alternative deposition techniques such as magnetron sputtering and pulsed laser deposition have been increasingly explored with the aim of fabricating innovative coatings and exhibiting higher mechanical and—eventually—better clinical performance [6].

Recently, we started investigating the deposition of functional nanostructured ceramic thin films by the pulsed electron deposition (PED) technology [7, 8]. Among the most useful features of PED technique are a high fidelity in the transfer of the stoichiometry of the deposition target to the film and the possibility to efficiently work also at room temperature (thus enabling to coat also heat-sensitive materials) [9, 10]. In a previous study, we reported, for the first time, the fabrication of nanostructured thin films with chemical composition and crystallinity very close to that of biogenic apatite, by simply using a fully deproteinized bovine bone shaft as a deposition target; in that study, we used a breakthrough upgrade of the PED technology, named Ionized Jet Deposition (IJD) [11]. The hypothesis behind this research is that biogenic apatite thin films, thanks to the great affinity in composition and crystallinity with natural bone apatite, will provide faster and more robust implant osseointegration, when compared to conventional HA coatings.

In the present study, we specifically investigated the adhesion, proliferation, and osteogenic differentiation of human dental pulp stem cells (hDPSCs) on such biogenic apatite films, in order to pave the way for future preclinical studies. The human dental pulp represents a very interesting stem cell source due to the low invasiveness of the procedures for cell isolation and to the high proliferation and multipotency of stem cells contained within this tissue [12]. As reported in the literature, hDPSCs have been extensively investigated for their characteristics and their ability to differentiate towards cell lineages derived from the three embryological layers, mostly due to their origin from the neural crest [13]. Several reports demonstrated that hDPSCs are able to commit towards osteogenic, adipogenic, and myogenic lineages [14, 15], together with being capable of differentiating towards glial and neuronal cells [16, 17] and into insulin-producing cells [18]. In this study, we evaluated whether and how this novel biogenic apatite films are able to influence the behavior of a subpopulation of hDPSCs, formerly immune selected for the expression of the surface markers STRO-1 and c-Kit, their morphology, proliferation, and osteogenic potential.

## 2. Materials and Methods

### 2.1. Film Deposition and Characterization

Biogenic apatite and HA thin films were deposited by the Ionized Jet Deposition (IJD) technology [19] (Noivion Srl, Rovereto, Italy), as described elsewhere [11]. Borosilicate microscope glass slides (2 × 2 cm, 1 mm of thickness) were used as a substrate after ultrasonic cleaning in isopropyl alcohol for 5 minutes and then in water for additional 5 minutes. After deposition, part of the samples was annealed at 400°C for 1 hour in air to increase the crystallinity of the films using a 20°C min^−1^ heating/cooling ramp.

Film morphology and surface roughness were investigated by atomic force microscopy (AFM) using a Stand-Alone SMENA AFM (NT-MDT, Moscow, Russia) operating in semicontact mode at ambient conditions. Surface roughness was expressed as root mean square (RMS) roughness and calculated by averaging the values obtained upon several nonoverlapping sample regions, whose dimensions ranged from 10 × 10 *μ*m^2^ to 2 × 2 *μ*m^2^. All images were unfiltered, except for a second order leveling, and acquired with a resolution of 512 × 512 pixels.

Surface wettability was estimated by water contact angle (CA) measurement using a Digidrop contact angle meter (GBX Instrumentation Scientifique, Romance, France), the volume of the drop being ~0.5 *μ*l. Contact angle values were acquired on at least three different positions over the sample surface, and the mean value was determined.

To evaluate the degree of adhesion of the films to the glass substrate, micro scratch tests were performed using a Micro-Scratch Tester (MST, CSM Instruments-Anton Paar Srl, Peseux, Switzerland), following the specifications of ISO 20502 for the determination of mechanical failure modes of ceramic coatings [20]. Briefly, a conical Rockwell C stylus with spherical apex indenter tip (angle 120°, sphere radius 100 *μ*m) was subjected to a progressive normal load from 0.01 N to 10 N and moved across the surface of the coated sample with a scan speed of 10 mm min^−1^ and a loading rate of 10 N min^−1^. A minimum of five scratches was carried out on each sample. The worn tracks were investigated by an optical microscope (20x and 50x zooms) mounted on the micro scratch platform to determine the failure modes of the coating and associate them with the load at which they occur. In general, a series of failure modes can be observed and used to study the mechanical behavior of the coated surface, where the onset of the *n*th failure mode defines the critical normal force Lcn.

### 2.2. Cell Isolation and Immune Selection

Human dental pulp was extracted from the third molars of adult subjects (*n* = 3) undergoing a routine tooth extraction, after written informed consent of the patients. Cells were isolated from the dental pulp as formerly described [15]. Briefly, the dental pulp was harvested from the teeth and immersed in a digestive solution (*α*-MEM plus 3 mg/mL type I collagenase and 4 mg/mL dispase) for 1 h at 37°C. Subsequently, the pulp was dissociated and filtered onto 100 *μ*m Falcon cell strainers, in order to obtain a cell suspension. Cell suspension was then plated in 75 cm^2^ flasks and maintained in a culture medium (*α*-MEM with 10% heat-inactivated foetal bovine serum (FBS), 2 mM L-glutamine, 100 U/mL penicillin, and 100 *μ*g/mL streptomycin), at 37°C and 5% CO_2_. Upon reaching confluence, cells underwent immune selection through MACS technology (Miltenyi Biotec): firstly, the cell fraction expressing STRO-1 surface antigen was isolated, by using a mouse anti-STRO-1 antibody (Santa Cruz), then positively sorted cells were replated and expanded in a culture medium. When confluence was reached, STRO-1^+^ hDPSCs underwent a further immune-selection, by using a rabbit anti-c-Kit antibody (Santa Cruz), in order to obtain the STRO-1^+^/c-Kit^+^ hDPSC population. About 5 × 10^6^ cells were used for each immune selection.

### 2.3. Evaluation of Cell Adhesion and Cell Proliferation

In order to determine which of the coatings can better promote adhesion and proliferation of STRO-1^+^/c-Kit^+^ hDPSCs, cells were seeded at the cell density of 2 × 10^3^ cells/cm^2^ on as-deposited (HA_AD and BAp_AD) and annealed (HA_HT and BAp_HT) HA and BAp films. Cells were monitored for 1 week, cell counting being performed everyday on 10 randomly chosen fields. After determining the most suitable film, the expression of STRO-1 and c-Kit stemness markers was investigated at 1 and 3 weeks of culture under standard conditions, by immunofluorescence analysis, as described in [16]. Briefly, monolayer cells were fixed with 4% paraformaldehyde (PFA) in phosphate-buffered saline (PBS) for 15 minutes at room temperature. Then, after removing PFA, cells were rinsed thrice with PBS, then saturated in a solution containing 3% BSA in PBS for 30 minutes at room temperature. Then, following washings in PBS, cells were incubated for 1 hour at room temperature with the following primary antibodies: mouse anti-STRO-1 and rabbit anti-c-Kit, all diluted 1 : 50 in PBS containing 3% BSA. Subsequently, cells were washed in PBS containing 3% BSA and incubated for 1 hour at room temperature with the secondary antibodies, diluted 1 : 200 in PBS containing 3% BSA: goat anti-mouse Alexa647 and goat anti-rabbit Alexa488 (Life Technologies). After washing in PBS, samples were stained with 1 mg/ml DAPI in PBS for 1 minute and then mounted with an antifading medium.

Furthermore, in order to evaluate whether the film may induce morphological changes in hDPSCs, fixed monolayer cells underwent immunolabeling with TRITC-conjugated anti-phalloidin antibody (Life Technologies).

For all tests, hDPSCs cultured under standard conditions on glass coverslips were used as controls.

### 2.4. Osteogenic Differentiation

In order to perform osteogenic differentiation, STRO-1^+^/c-Kit^+^ hDPSCs were seeded at approximately 2.5 × 10^3^ cells/cm^2^ on a BAp_HT film and on standard glass coverslips. Upon reaching confluence, the culture medium was replaced with osteogenic medium (culture medium supplemented with 100 *μ*M 2P-ascorbic acid, 100 nM dexamethasone, and 10 mM b-glycerophosphate) [17]. Osteogenic induction was performed for 3 weeks, then the expression of typical differentiation markers, such as Runx-2, osterix (OSX), and osteocalcin (OCN), was evaluated by immunofluorescence and Western blot analyses.

STRO-1^+^/c-Kit^+^ hDPSCs seeded on BAp films not supplied with osteogenic medium and hDPSCs cultured on glass coverslips or plastic tissue culture dishes were used as controls.

### 2.5. Western Blot

Whole cell lysates were obtained from undifferentiated STRO-1^+^/c-Kit^+^ hDPSCs and STRO-1^+^/c-Kit^+^ hDPSCs differentiated toward osteogenic lineage, either on BAp_HT films and on glass coverslips, after 3 weeks of induction. Cells were harvested, washed with PBS, and gently lysed on ice for 10 minutes in hypotonic lysis buffer (30 mM Tris-HCl, pH 7.8, containing 1% Nonidet P40, 1 mM EDTA, 1 mM EGTA, 1 mM Na_3_VO_4_, and protease and phosphatase inhibitors cocktail; Sigma-Aldrich). After brief sonication and microcentrifugation at 12,000 rpm for 15 minutes at 4°C, the supernatants were collected and protein concentrations were determined by Bradford assay. Thirty *μ*g of total proteins from each sample was separated by sodium dodecyl sulfate-polyacrylamide gel electrophoresis (SDS-PAGE) and then transferred to PVDF membranes. Membranes were incubated overnight at 4°C with rabbit anti-Runx2 and mouse anti-OCN (Abcam; diluted 1 : 1000 in Tris-buffered saline Tween 20 plus 2% BSA and 3% nonfat milk). Horseradish peroxidase-conjugated anti-rabbit and anti-mouse secondary antibodies diluted 1 : 3000 were then incubated for 30 minutes at room temperature. All membranes were visualized using Enhanced Chemiluminescence (Amersham). Anti-actin antibody was used as control of protein loading. Densitometry was performed on three independent experiments by Fiji ImageJ software. An equal area was selected inside each band and the integrated density was calculated. Data were normalized to values of background and of control actin band. Values were expressed as mean ± SD.

### 2.6. Statistical Analysis

All experiments were performed in triplicate. Data were expressed as mean ± standard deviation (SD). Differences between two experimental conditions were analyzed by paired, Student's *t*-test. Differences among three or more experimental samples were analyzed by ANOVA followed by Newman-Keuls post hoc test (GraphPad Prism Software version 5 Inc., San Diego, CA, USA). In any case, significance was set at *P* < 0.05.

## 3. Results

### 3.1. Film Characterization

**Figure 1:**
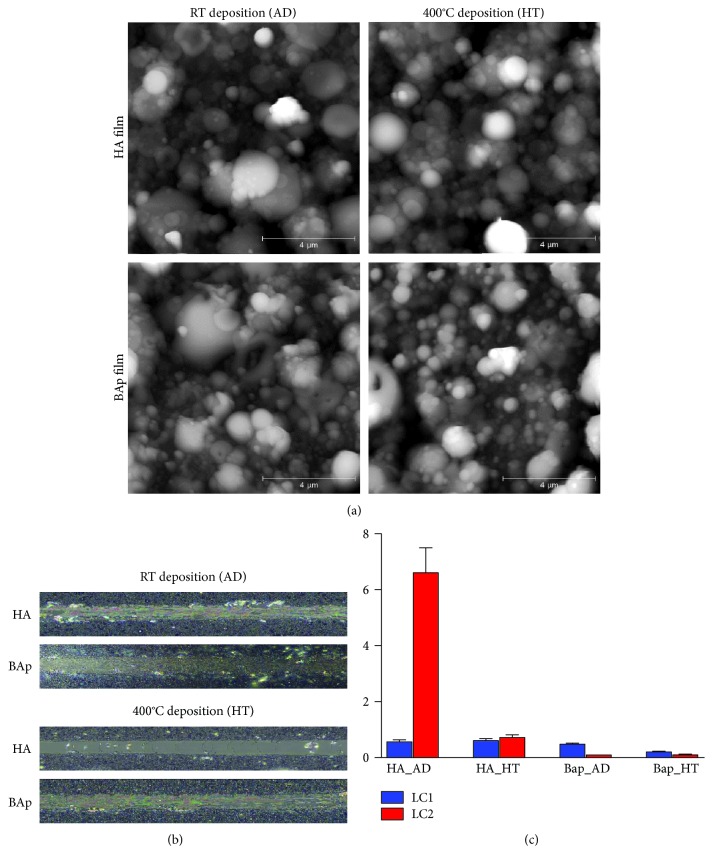
(a) Atomic force microscopy images of the surface of as-deposited and annealed HA and BAp thin films obtained by PED. Worn tracks from 0 to 2 N (b) and critical loads LC1 (initial delamination) and LC2 (complete delamination) of HA and BAp films (c).

Surface topography was investigated by AFM. Both films deposited from biogenic apatite or HA targets exhibited a similar micro/nanostructured granular surface, with a grain size ranging from about 100 nanometers for smaller particulate to a few microns for larger aggregates (Figure 1(a)). The thermal annealing process did not substantially modify the surface topography, in agreement with previous data [11]. Nevertheless, surface roughness was slightly higher for BAp films compared to HA films, as reported in Table 1. The surface of BAp films was found to be more hydrophilic than corresponding HA films. Upon thermal treatment, the contact angle values decreased for both BAp and HA to a similar extent; thus, all the films increased their hydrophilicity upon annealing. Film adhesion to the glass substrate has been evaluated by micro scratch tests (Figures 1(b) and 1(c)).

As a general trend, BAp films better adhered to the glass substrate compared to HA films, as indicated by the absence of complete delamination (Figure 1(c)) at the maximum load applied (i.e., 10 N), which was instead detectable for HA films. Among the investigated samples, HA_HT showed the lowest adhesion degree, as suggested also by the complete exposure of the glass substrate at extremely low loads (Figure 1(b)). On the contrary, BAp_HT showed the highest adhesion degree, as indicated by the lower amount of film removed from the micro scratch indenter tip and deposited beside the worn track compared to BAp_AD. This fact clearly indicates a significant improvement of the mechanical adhesion of the BAp film to the substrate upon annealing, as a consequence of increased crystallinity [11].

### 3.2. Cell Adhesion and Cell Proliferation

**Figure 2:**
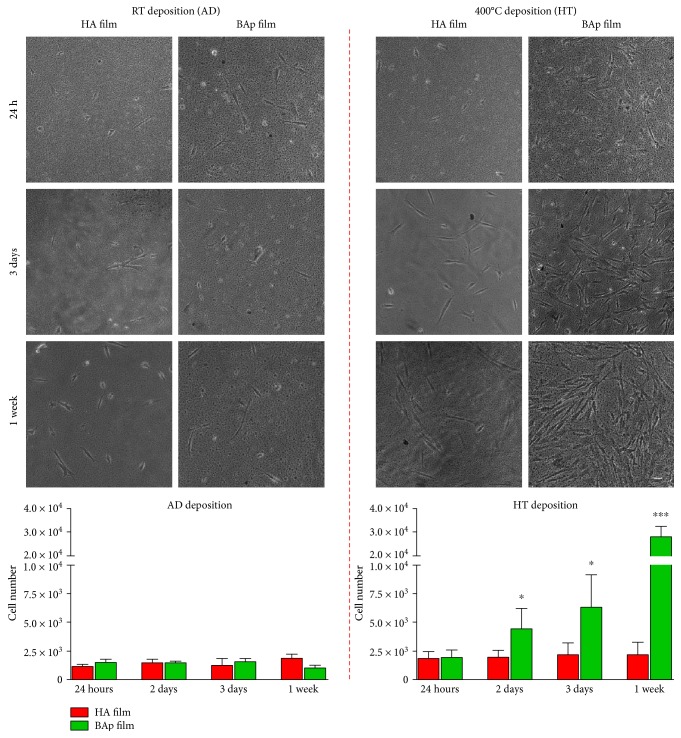
Representative phase contrast images showing hDPSC adhesion after 24 h of culture and cell proliferation on HA and BAp films after room temperature deposition (AD) and after 400°C deposition (HT) up to one week. Histograms represent the growth kinetics of hDPSCs cultured on different films at different time points. ^∗^*P* < 0.05, ^∗∗∗^*P* < 0.001 hDPSCs cultured on BAp_HT versus hDPSCs cultured on HA_HT films. Scale bar: 10 *μ*m.

After cell seeding on HA and BAp films, cell adhesion and cell proliferation were evaluated at different time points. No significant differences were observed in cell adhesion after 24 hours of culture on HA and BAp films, as shown by phase contrast images reported in Figure 2.

At the same time, the proliferation of STRO-1^+^/c-Kit^+^ hDPSCs cultured on HA_AD and BAp_AD did not show differences through 1 week of culture, as reported in the histograms (Figure 2). Interestingly, significant differences were observed, instead, when analyzing cell proliferation of hDPSCs cultured on BAp_HT, in comparison with the counterpart cultured on HA_HT. In particular, statistically significant values of cell growth were measured on day 2 and 3, up to a peak value at day 7 (Figure 2; ^∗^*P* < 0.05, ^∗∗∗^*P* < 0.001 versus HA_HT).

**Figure 3:**
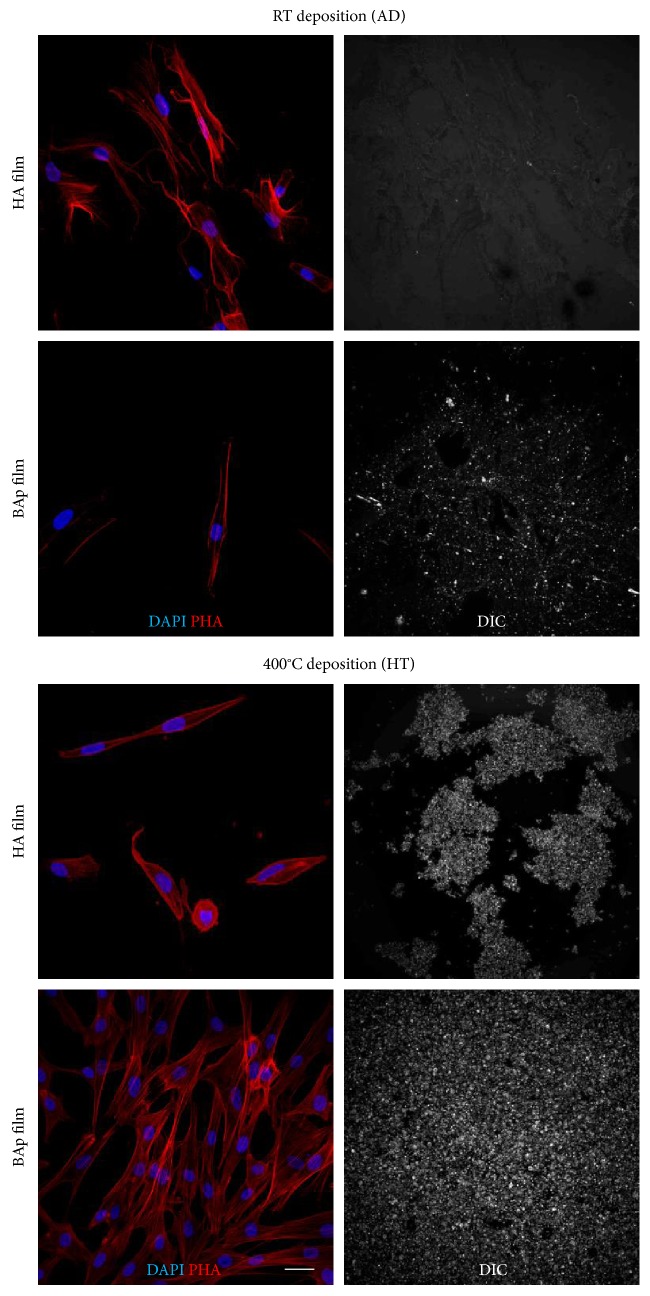
Representative immunofluorescence images showing cell morphology of hDPSCs cultured on HA and BAp films and DIC images representing film adhesion to the underlying surface at 7 days of culture. Immunofluorescence analysis was performed against phalloidin. Nuclei were counterstained with DAPI. Scale bar: 10 *μ*m.

Immunolabeling with TRITC-conjugated anti-phalloidin antibody revealed hDPSC morphology following adhesion to the different films (Figure 3). Their typical fibroblast-like morphology was completely preserved when cells were grown on BAp_HT film. At the same time, hDPSCs showed a spread homogeneous proliferation through the whole BAp_HT film area, when compared to the other films. As highlighted by DIC images, a well-preserved adhesion to the underlying surface was observed for BAp_HT film, whereas film adhesion was only partially maintained in HA_HT counterpart, barely detectable in BAp_AD films and completely lost in HA_AD.

Based on these preliminary data, only BAp_HT films were further characterized.

### 3.3. Cell Morphology and Stemness Marker Expression

**Figure 4:**
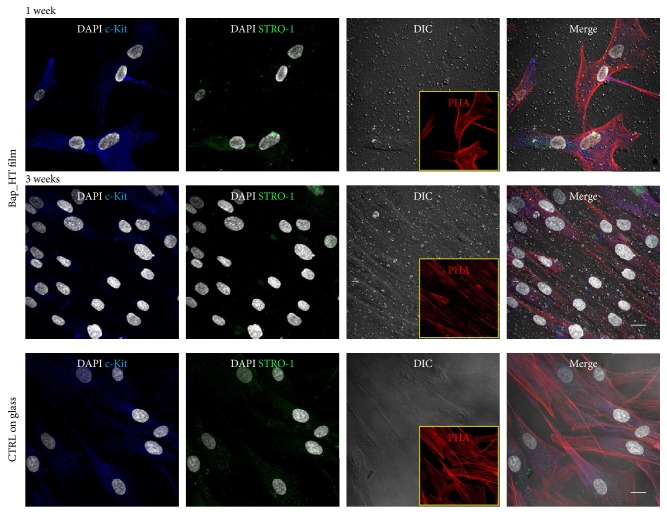
Evaluation of stemness marker expression by hDPSCs grown on BAp_HT films at 1 and 3 weeks of culture. DIC images show the adhesion of the films to the underlying surface and the presence of hDPSCs. Inserts reporting phalloidin labeled cells represent cell morphology of hDPSCs cultured on BAp_HT films after 1 and 3 weeks of culture. Nuclei were counterstained with DAPI. Scale bar: 10 *μ*m.

In order to evaluate the expression of STRO-1 and c-Kit markers in hDPSCs cultured on BAp_HT film, immunofluorescence analysis was performed after 1 and 3 weeks of culture.  Figure 4 shows that BAp_HT film did not affect the expression of the stemness markers STRO-1 and c-Kit after 1 week, whereas a slight decrease in the expression of both the surface markers was detected by confocal analysis after 3 weeks of culture. No significant differences in the expression of STRO-1 and c-Kit were observed in hDPSCs cultured on control glass coverslips. Phalloidin immunolabeling also demonstrated that fibroblast-like morphology of hDPSCs cultured on BAp_HT film was similar to hDPSCs grown on control glass coverslips.

### 3.4. Evaluation of Osteogenic Differentiation

In order to assay the effect of BAp_HT film on osteogenic differentiation potential of STRO-1^+^/c-Kit^+^ hDPSCs, immunofluorescence analysis was performed on cells after 3 weeks of expansion under standard culture conditions and after 3 weeks of induction in osteogenic medium.

**Figure 5:**
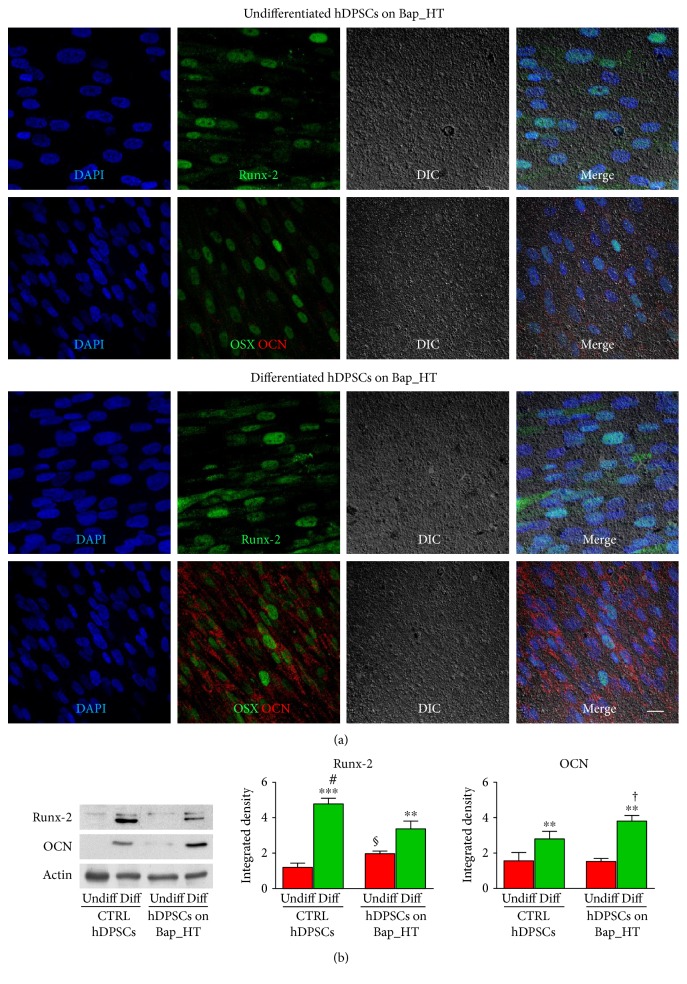
(a) Immunofluorescence analysis performed on undifferentiated hDPSCs and on osteogenic differentiated hDPSCs cultured on BAp_HT films for 3 weeks in standard culture conditions and osteogenic medium, respectively. Representative images show the expression and cellular localization of Runx-2, OSX, and OCN. DIC images report film adhesion at 3 weeks of culture and hDPSC distribution on the film surface. Nuclei were counterstained with DAPI. Scale bar: 10 *μ*m. (b) Western blot analysis was performed on undifferentiated and osteogenic differentiated hDPSCs grown on control glass coverslips and on BAp_HT films, respectively, for Runx-2 and OCN expression. Histograms represent densitometry analysis of Runx-2 and OCN. Actin bands were used as loading control. ^∗∗^*P* < 0.01, ^∗∗∗^*P* < 0.001 differentiated hDPSCs versus undifferentiated hDPSCs grown on the two different surfaces; ^#^*P* < 0.05 differentiated hDPSCs on control coverslips versus differentiated hDPSCs on BAp_HT film; ^§^*P* < 0.05 undifferentiated hDPSCs on BAp_HT films versus undifferentiated hDPSCs on control coverslips; ^†^*P* < 0.05 differentiated hDPSCs on BAp_HT films versus differentiated hDPSCs on control coverslips.

Under standard conditions, hDPSCs expressing STRO-1 and c-Kit markers showed high levels of the osteogenic markers Runx-2 and OSX, whereas only a slight staining against the later osteogenic marker OCN was revealed (Figure 5(a)). Following induction with osteogenic medium for 3 weeks, besides Runx-2 and OSX expression, also high levels of OCN were observed. Western blot analysis revealed that hDPSCs cultured in osteogenic medium, either on control coverslips or BAp_HT films, showed statistically significant higher levels of Runx-2 and OCN when compared to undifferentiated hDPSCs (^∗∗^*P* < 0.01, ^∗∗∗^*P* < 0.001 versus undifferentiated hDPSCs). Interestingly, hDPSCs cultured in an osteogenic medium on control glass coverslips expressed higher levels of Runx-2 (^#^*P* < 0.05), compared to hDPSCs differentiated on BAp_HT films, as demonstrated by densitometry analysis (Figure 5(b)). Moreover, only undifferentiated hDPSCs grown on BAp_HT film expressed higher levels of Runx-2, with respect to undifferentiated hDPSCs grown on control coverslips (^§^*P* < 0.05). This might be due to the ability of the BAp_HT film to trigger a preliminary commitment of hDPSCs towards the osteogenic lineage, even under standard culture conditions. Besides, when differentiated on BAp_HT film, hDPSCs showed higher levels of OCN, compared to hDPSCs differentiated on control coverslips (^†^*P* < 0.05 versus hDPSCs differentiated on control coverslips).

## 4. Discussion

Based on the “biomimetic principle” [21], coatings made of fully biomimetic apatite with suitable mechanical strength, in virtue of higher chemical/structural affinity with the inorganic phase of bone, are expected to boost implant osseointegration when compared to nonbiomimetic apatite, other calcium phosphate phases, or different bioceramic coatings. In this study, we demonstrated that, indeed, nanostructured biogenic apatite thin films deposited by the novel IJD technique promoted the proliferation of a subpopulation of hDPSCs expressing STRO-1 and c-Kit, when compared to HA coatings. Besides, cell proliferation on HA coatings and as-deposited BAp films was limited.

It is widely reported that surface roughness and hydrophilicity/hydrophobicity can strongly affect cell adhesion and proliferation [22]. In particular, coatings showing a fine, nanostructured superficial texture, as the ones deposited in this study, are expected to enhance cell adhesion and spreading, thanks to their higher surface area compared to microstructured coatings, thus eventually promoting bone tissue regeneration [23]. In addition, for what the wettability of the surface is concerned, moderate or highly hydrophilic surfaces, as one of the apatite films deposited here, have been demonstrated to promote cell adhesion and proliferation compared to highly hydrophobic surfaces [24, 25].

Another key parameter regarding thin films is the degree of adhesion to the substrate, as a stable and well-adherent film will provide a suitable platform for cell anchoring and spreading, while a fast degrading or poor-adherent film will represent an instable surface impairing cell adhesion.

In the present study, both surface roughness and wettability of BAp films were slightly higher than those of HA films. However, these small differences cannot justify alone the trend of hDPSC adhesion and proliferation observed in this study. In fact, if that was the case, BAp_AD and BAp_HT should have behaved similarly, while, instead, the proliferation on BAp_HT was statistically increased on BAp_HT compared to BAp_AD. This fact, together with the confocal images of the residual films at day 7, which pointed out that only BAp_HT showed an intact film at that experimental time, indicate that the different adhesion and proliferation of hDPSCs are to be attributed to the very different adhesion degree observed among the films. In fact, as reported in Figure 3, the DIC images of the different films' deposition clearly showed that after one week of culture only the BAp_HT film was still present and uniformly adherent to the underlying surface, whereas the other films lost their adhesion to the surface. This fact could explain the differences regarding the cell proliferation on the other films. Based on this preliminary data, our study was focused on BAp_HT film. As a matter of fact, the deposition of BAp_HT film was maintained through culture time, either in expansion medium and after 3 weeks of induction in osteogenic medium, suggesting that this film is firmly stable, which is a desirable requirement for the success of regenerative medicine applications, such as orthopedic and craniofacial implants.

The adhesion of the film on the underlying stage might have influenced not only cell adhesion but also the proliferation ability of STRO-1^+^/c-Kit^+^ hDPSCs. Indeed, stem cells showed an increasing trend in growth kinetics, when cultured on BAp_HT films, resembling the cell proliferation rate observed during culture on control glass coverslips.

Moreover, hDPSCs preserved their typical fibroblast-like morphology and maintained the expression of stemness markers after 7 days of culture. Conversely, at 3 weeks of culture on BAp_HT film, hDPSCs showed a slightly decreased expression of STRO-1 and c-Kit markers and, in parallel, an appreciable immunolabeling against the osteogenic markers Runx-2 and OSX. In particular, the differentiation behavior of hDPSCs was positively affected by BAp film, which was able to promote a spontaneous commitment towards osteogenic lineage, without the addition of osteogenic medium, and favored an earlier induction of osteogenic differentiation, as demonstrated by lower levels of Runx2 and higher levels of OCN, after 3 weeks of culture in an induction medium, when compared to hDPSCs cultured under standard conditions. This data was confirmed by immunofluorescence analysis that clearly showed the expression and the cellular localization of osteogenic markers on BAp_HT film, in standard culture medium and after 3 weeks of osteogenic induction.

## 5. Conclusions

The results reported in this work suggested that biogenic bone apatite thin films obtained by IJD technique and subsequently annealed offer a suitable substrate for hDPSC adhesion, proliferation, and commitment towards osteogenic differentiation. Further investigations of the biological effects of BAp_HT films through *in vivo* studies will establish the real bone regenerative potential for both dental and orthopedic applications.

## Figures and Tables

**Table 1:** Root mean square (RMS) roughness and wettability of deposited films and glass substrate.

Sample	RMS at 10 *μ*m^2^ (nm)	Contact angle (°)
HA_AD	140 ± 19	57.9 ± 2.8
HA_HT	173 ± 9	48.8 ± 3.1
BAp_AD	243 ± 46	47.6 ± 1.4
BAp_HT	209 ± 24	36.2 ± 0.9
Glass substrate	1.0 ± 0.1	24.1 ± 3.1
