# Corrigendum to “Osteogenic Differentiation of hDPSCs on Biogenic Bone Apatite Thin Films”

**DOI:** 10.1155/2017/6587384

**Published:** 2017-12-21

**Authors:** Michele Bianchi, Alessandra Pisciotta, Laura Bertoni, Matteo Berni, Alessandro Gambardella, Andrea Visani, Alessandro Russo, Anto de Pol, Gianluca Carnevale

**Affiliations:** ^1^Rizzoli Orthopaedic Institute, NanoBiotechnology Laboratory, Via di Barbiano 1/10, 40136 Bologna, Italy; ^2^Department of Surgery, Medicine, Dentistry and Morphological Sciences, University of Modena and Reggio Emilia, Via del Pozzo 71, 41124 Modena, Italy; ^3^Rizzoli Orthopaedic Institute, Laboratory of Biomechanics and Technology Innovation, Via di Barbiano 1/10, 40136 Bologna, Italy

In the article titled “Osteogenic Differentiation of hDPSCs on Biogenic Bone Apatite Thin Films” [1], the second affiliation was incorrect. The corrected affiliations are shown above.
